# Book Reviews

**Published:** 1874-11-15

**Authors:** 


### Book Reviews.

[*Received through W. B. Keen, Cooke & Co.*]

CROUP: IN ITS RELATIONS TO TRACHEOTOMY. By J. Solis Cohen, M.D. Philadelphia: Lindsay & Blakiston.

This essay, read before the Philadelphia County Medical Society, and now reprinted from their transactions, forms a pamphlet of seventy-five pages.

THERAPEUTICS AND MATERIA MEDICA. A Systematic Treatise on the Action and Uses of Medicinal Agents. By Alfred Stille, M.D. Fourth edition; thoroughly revised and enlarged. Philadelphia: H. C. Lea, 1874.

This work has long been recognized in this country as the leading and standard text book on materia medica. The present new edition comes to us thoroughly revised and somewhat enlarged. Some of the chapters have been entirely re-written. Several new articles have been introduced, and the nomenclature throughout has been brought into conformity with the last edition of the Pharmacopœia.

---

PHYSICIANS' VISITING LIST FOR 1875. Lindsay & Blakiston. Philadelphia.

We acknowledge the receipt of this deservedly popular visiting list for 1875, the twenty-fourth year of its publication.

---

The Chicago Medical Register and Directory for 1874. Chicago: W. B. Keen, Cooke & Co.

We have already expressed our opinion very freely and fully regarding this work.

---

[*Received through Messsrs. Jansen, McClurg & Co.*]

ESSENTIALS OF THE PRINCIPLES AND PRACTICE OF MEDICINE. A Hand Book for Students and Practitioners. By Henry Hartshorn, A.M., M.D. Fourth edition. Philadelphia: H. C. Lea.

This little work is already familiar through its former editions. As a brief, condensed, but comprehensive hand book, it cannot be improved upon.

---

CLINICAL LECTURES ON DISEASES OF THE NERVOUS SYSTEM. By Wm. A. Hammond, M.D. Reported and edited by T. M. B. Cross, M.D. New York: D. Appleton & Co.

This is a volume of 287 octavo pages. The twenty lectures of which it is constituted embrace the following topics: partial cerebral anæmia, alternate, or cross hemiplegia, congestion of the spinal cord, lead paralysis, chorea, aphasia, facial paralysis, glosso-labio laryngial paralysis, cerebral hæmorrhage, posterior spinal sclerosis, progressive muscular atrophy, convulsive tremor, chronic basilar meningitis, cerebral congestion, epilepsy, facial neuralgia, cervico-occipital and intercostal neuralgia, sciatica, organic infantile paralysis.

The peculiar views and teachings of Prof. Hammond have already become familiar to the profession through his larger and more comprehensive treatise.

---

CLINICAL LECTURES ON VARIOUS IMPORTANT DISEASES. By Nathan S. Davis, A.M., M.D. Second edition. Philadelphia: H. C. Lea.

In this second edition two new lectures have been added, one on *mania a potu* and chronic diseases of the brain, the other on pneumonia.

---

THE CHICAGO JOURNAL OF NERVOUS AND MENTAL DISEASE. Edited by J. S. Jewell, M.D., and H. M. Bannister, M.D.

The number for October, which is just issued, completes the first volume of this new quarterly.

Each of the four numbers has been filled with the most valuable and important matter relating to this specialty. A full supply of original communications and translations has been given, while the department of reviews and gleanings has comprised a complete epitome of all the foreign and home literature relating to this department. Altogether the complete and thorough manner in which they have carried out their enterprise reflects the highest credit upon the energy and talent of the editors.

The editors announce that the *Journal* has met with favor and patronage largely exceeding their expectations, and, "that there has not been, and is not now, a thought of its discontinuance, whatever changes the future may bring."

